# Evaluation of In Vitro Phototoxicity of a Minibody-IR700 Conjugate Using Cell Monolayer and Multicellular Tumor Spheroid Models

**DOI:** 10.3390/cancers13133356

**Published:** 2021-07-04

**Authors:** Mouldy Sioud, Petras Juzenas, Qindong Zhang, Andrius Kleinauskas, Qian Peng

**Affiliations:** 1 Division of Cancer Medicine, Department of Cancer Immunology, Oslo University Hospital, University of Oslo, Ullernchausseen 70, 0379 Oslo, Norway; Qindong.Zhang@rr-research.no; 2 Division of Laboratory Medicine, Department of Pathology, Oslo University Hospital-Radiumhospitalet, Ullernchausseen 70, 0379 Oslo, Norway; Petras.Juzenas@rr-research.no (P.J.); Andrius.Kleinauskas@rr-research.no (A.K.); Qian.Peng@rr-research.no (Q.P.)

**Keywords:** photodynamic therapy, photoimmunotherapy, antibodies, photosensitizers, antibody-drug conjugates

## Abstract

**Simple Summary:**

While photodynamic therapy (PDT) has emerged as an attractive treatment for certain cancer types, it still lacks cancer specificity, which limits therapeutic efficacy and damages normal tissues. The present study aimed to evaluate the targeting potential of a novel minibody that recognizes a cell surface receptor expressed on various cancer cell lines. The engineered minibody-photosensitizer conjugate (MS5-IR700) killed target cells in both cell monolayer and tumor spheroid cultures. Additionally, the conjugate induced immunogenic cell death. Hence, the developed minibody could be used as a photosensitizer carrier for -cancer cell-targeted PDT.

**Abstract:**

Photodynamic therapy (PDT) is a treatment strategy that utilizes photosensitizers (PSs) and light of a specific wavelength to kill cancer cells. However, limited tumor specificity is still a drawback for the clinical application of PDT. To increase the therapeutic efficacy and specificity of PDT, a novel human minibody (MS5) that recognizes a cell surface receptor expressed on various cancer cells was labeled with the hydrophilic phthalocyanine PS IR700 to generate an MS5-IR700 conjugate that is activated by near-infrared (NIR) light. The phototoxicity of the conjugate was mainly tested against the PC3 prostate cancer cell line. The MS5-IR700 conjugate killed PC3 cells after NIR light irradiation as compared to untreated cells or cells treated with IR700 alone. Time-course analysis of cell viability revealed a high percentage of cell death during the first hour in PC3 cells exposed to the MS5-IR700 conjugate and NIR light irradiation. After irradiation, the MS5-IR700 conjugate-treated PC3 cells displayed cellular swelling, round shape, and rupture of the cell and nuclear membranes. In a co-culture model, the MS5-IR700 conjugate killed MS5-positive Ramos lymphoma cells specifically, while leaving MS5-negative cells unaffected. In line with the data obtained with the monolayer cultures, the MS5-IR700 conjugate also killed PC3 cancer cell spheroids. The treatment induced relocation of heat shock protein 70 and calreticulin to the cell surface, implying the induction of immunogenic cell death. Overall, the data suggest that the developed MS5-IR700 conjugate is a promising therapeutic agent that warrants further preclinical studies.

## 1. Introduction

Photodynamic therapy (PDT) is a two-stage strategy involving the administration of a photosensitizer (PS) followed by exposure to light [1,2,3,4,5]. In the presence of tissue oxygen, the PS activated by light of a specific wavelength can stimulate the production of reactive oxygen species (ROS), leading to cell death by apoptosis or necrosis. Today, PDT is used in the clinical treatment of several cancers, including skin, esophageal non-small cell, and oral cancers [3,5,6]. Although the light activation of PSs enables a certain level of selectivity and prevents damage to healthy tissue surrounding the tumor, photosensitive reactions are regularly seen in normal tissues, such as the skin and eyes that are exposed to daylight [7]. For example, patients with esophageal cancer who are treated with PDT using talaporfin sodium (Laserphyrin^®^) are informed to avoid direct sunlight for 2 weeks [5]. Additionally, most porphyrins used as PSs are hydrophobic and tend to aggregate after intravenous administration, resulting in inefficient delivery to tumor tissues [3]. The major remaining challenge is thus to identify a ligand that can navigate PSs to tumor sites.

To increase specificity, certain photosensitizers have been conjugated to antibodies or antibody fragments targeting receptors overexpressed on tumor cells or tumor vasculatures [8,9,10,11,12,13,14,15], a therapeutic option known as photoimmunotherapy (PIT, 9). The engineered agents benefit from the targetable property of antibodies but rely on the cytotoxicity that is triggered through ROS when the PS is photoactivated. While the generated conjugates showed promising killing efficacy both in vitro and in vivo against tumor cells [6,8], it is still unclear which antibody format to be used to maximize the therapeutic efficacy and specificity [3,6,9,16,17]. Considering the importance of establishing new therapeutic options for cancer patients, the development of new antibodies that target common receptors expressed on tumor cells may render immunotherapy more effective and available to more patients including those who did not respond to current therapies [18]. By use of phage antibody libraries, we have discovered a number of human scFv antibody fragments that recognize cell surface receptors expressed on various types of cancer [19,20,21]. One of the selected scFv fragments was converted into a human minibody (scFv-Fc fusion protein, named MS5) and found to inhibit tumor growth [20]. The minibody binding receptor seems to be overexpressed by most malignant cells when compared to their normal counterparts. For example, only a weak binding was observed with normal B cells when compared to B cell lymphoma cell lines [20]. As a first step towards the development of small photoimmunoconjugates, here the photosensitizer IR700 was conjugated to the MS5 minibody and the phototoxicity of the conjugate was investigated using monolayer and spheroid cultures. The engineered conjugate efficiently and specifically killed cancer cells in both monolayer and tumor spheroid cultures.

## 2. Materials and Methods

### 2.1. Cell Lines and Reagents

PC-3 (ATCC^®^ CRL-1435) prostate, MDA-MB-453 (ATCC^®^ HTB-131) breast, SKOV-3 (ATCC^®^ HTB-77) ovarian, U87MG (ATCC^®^ HTB-14) glioma, SW900 (ATCC^®^ HTB-59) lung, Ramos (RA1) (ATCC^®^ CRL-1596) lymphoma, and KG1a (ATCC^®^ CCL-246.1) leukemia cell line were purchased from American Type Culture Collection (Manassas, VA, USA). The human head and neck carcinoma cell line scc-U8 was kindly provided by Dr. Robinson (Erasmus MC, Rotterdam, The Netherlands). The cells were cultured in either RPMI 1640 or DMEM medium supplemented with 10% heat-inactivated fetal bovine serum (FBS) and 1% penicillin-streptomycin. Cells were cultured at 37 °C in 5% CO_2_. The water-soluble phthalocyanine dye IRDye 700DX NHS ester was purchased from LI-COR Bioscience (Lincoln, NE, USA). FITC-conjugated anti-Human IgG (Fc specific) antibody was purchased from Sigma (St. Louis, MO, USA), anti-HSP70 antibody (1H1) was purchased from Stress Marq (Biosciences, Cadboro Bay, BC, Canada, c), and anti-Calreticulin antibody (FMC 75) was purchased from Abcam (Cambridge, UK). Propidium iodide, fluorescein diacetate (FDA), and 2′,7′-dichlorodihydro-fluorescein diacetate (H2DCF-DA) were purchased from Invitrogen (Carlsbad, CA, USA). All other chemicals were of reagent grade.

### 2.2. Conjugation of IR700 to MS5 Minibody

The MS5 minibody (1 mg) was incubated with IR700DX NHS ester (50 μg) in 1 mL 100 mM Na_2_HPO_4_, pH 8.5 at RT for 1 h with gentle rotation. The reaction was quenched with the addition of 100 μL 250 mM glycine buffer (pH 7.5) followed by the capture of the MS5-IR700 conjugate with protein A/G Plus agarose (Santa Cruz Biotechnology, Dallas, TX, USA). The agarose beads were washed 5 times in PBS buffer to remove unconjugated free IR700 molecules. Bound MS5-IR700 molecules were acid eluted by adding 200 μL of elution buffer (0.2 M glycine buffer, pH 2.5) and immediately neutralized with 30 μL Tris-Base buffer (1 M, pH 9) to obtain a pH around 7.5. The protein concentration was determined with Coomassie Plus assay kit (Pierce Biotechnology; Rockford, IL, USA) or Nanodrop. The amount of conjugated IR700 was determined by absorption at 690 nm using a Synergy LX spectrophotometer (BioTek, Agilent, Winooski, VT, USA). Under our experimental conditions each MS5 minibody molecule bound on average 1 to 2 IR700 molecules. SDS-PAGE was performed as a quality control for the conjugate.

### 2.3. Photocytotoxicity

Cancer cells were seeded in the 96-well flat-bottomed plates at 2 × 10^4^ cells per well in complete medium and incubate at 37 °C. After one day, the cells were incubated with the test molecules for 1 h at 4 °C, RT, or 37 °C. After washing with serum-free medium, 100 μL complete medium was added and then the cells were exposed to near-infrared (NIR). For this purpose, an in-house-built lamp consisting of an array of 24 light-emitting diodes (LED, 690 nm) was used. The power density of the LED array at the surface of the culture plate was 17 mW/cm^2^. Various light doses (5–60 J/cm^2^) were used. After light irradiation, the cells were placed in the incubator (dark) for various time periods, and cytotoxicity was measured using the CellTiter 96 AQueous One Solution Cell Proliferation Assay according to the manufacturer’s instructions (Promega, Madison, WI, USA). Optical densities were measured at 492 nm using a 96-well plate reader (TECAN, Sunrise; Männedorf, Switzerland). Plates that were not NIR-irradiated were shielded from ambient light. In some experiments, fluorescein diacetate (FDA) was used in combination with propidium iodide (PI) to stain live and dead cells, respectively.

### 2.4. ROS Production

To detect cellular production of reactive oxygen species (ROS), we have used the non-fluorescent reagent 2’,7’-dichlorodihydrofluorescein diacetate (H_2_DCFDA, Invitrogen). In the presence of ROS, H_2_DCFDA is converted to a fluorescent probe (DCF) that can be monitored by flow cytometry. PC3 cells were labeled with H2DCFDA (5 μM) in serum-free medium for 30 min at RT. After the cells were washed, resuspended in a complete medium, plated in a 48-well plate (1 × 10^5^ cells/200 μL/well), and then incubated with the MS5-IR700 conjugate (5 μg/mL) for 1 h at RT. After washing, a complete medium (200 μL/well) was added followed by NIR light irradiation (35 J/cm^2^). After 6 h or overnight incubation at 37 °C, the cells were harvested and analyzed by flow cytometry.

### 2.5. 3D Spheroid Cultures

PC3 cells growing in T25 flasks were treated with trypsin-EDTA for 5 min at 37 °C. Trypsin was then inactivated by adding 10 mL complete medium and the cells were pellet by centrifugation. The cell pellet was resuspended in a 5 mL complete medium and the cell number was determined. Five to ten thousand cells in 250 μL complete medium were plated in 96-well plates pre-coated with 1% agarose in sterile distilled water (50 μL/well). The optimal cell density was determined in preliminary spheroid generation tests. The plates were incubated at 37 °C for 24 h prior to the experiments. The formation of spheroids was verified using an inverted light microscope. The spheroids (200–300 μm in diameter) were treated with the MS5-IR700 conjugate (10 μg/mL) for 6 h at 37 °C. Subsequently, the plates were exposed to light (60 J/cm^2^), and image acquisition was performed once a day for 5–7 days. For viability measurement, the cells were incubated with propidium iodide (PI) for 5 min and then fluorescein diacetate (FDA) for 2 min prior to imaging.

### 2.6. Flow Cytometry

Flow cytometry was used to investigate the binding of the MS5-IR700 conjugate to cancer cells. Briefly, the cells (2 × 10^5^ cells/100 µL/sample) were incubated with or not with the MS5-IR700 conjugate (5 μg/mL) in FACS buffer (PBS with 1% BSA) for 40 min at 4 °C. After washing, the samples were analyzed by flow cytometry using a Cytoflex S cytometer (Beckman Coulter Life Sciences, Indianapolis, IN, USA) with the Cytexpert 2.1 software (Beckman Coulter). To monitor the expression of HSP70 and CRT after PDT treatment, the cells were stained with anti-HSP70 or anti-CRT specific monoclonal antibody antibodies, and then anti-mouse IgG-FITC was used for detection. Samples were analyzed on a BD FACS Canto II using the BD FACSDiva™ software (BD Biosciences, San Jose, CA, USA). All flow cytometry data were analyzed by FlowJo version 7.6.1 (FlowJo LCC, Ashland, OR, USA).

### 2.7. Bright-Field and Fluorescence Imaging

An inverted Zeiss Axiovert 40CFL microscope (Carl Zeiss AG, Jena, Germany) was used to image cells exposed to various treatments. Cells growing in 96-well plates were imaged with a 40× objective (LD A-Plan 40×/0.50 Ph2 Zeiss), while spheroids growing in 96-well plates were imaged with a 10× objective (A-Plan 10×/0.25 Ph1 Zeiss). For fluorescence images, excitation light from a 50 W super-pressure mercury lamp was used together with an appropriate beam splitter and filter combinations. For red fluorescence (PI), a beam splitter (filter set number 00, Carl Zeiss, AG, Jena, Germany) with an excitation bandpass filter 530–585 nm and emission long-pass filter 615 nm were used. For green fluorescence (FDA), a beam splitter (filter set number 09, Carl Zeiss) with an excitation bandpass filter 450–490 nm and emission long-pass filter 515 nm were used. All data were acquired and analyzed using the Carl Zeiss AxioVision software, version 4.8.2. (Carl Zeiss MicroImaging GmbH, Oberkochen, Germany)

### 2.8. Confocal Microscopy Imaging

PC3 cells were cultured in Lab-Tek chamber slides (Nalge Nunc International, Naperville, IL, USA) for 24 h, washed with serum-free medium, and then incubated with the MS5-IR700 conjugate for 1 h at RT in RPMI medium supplemented with 5% FBS. After incubation, the cells were washed and then incubated at 37 °C for one hour to allow receptor internalization. Subsequently, the cells were incubated with Hoechst 33342 (1 μg/mL) for 5 min, washed, and then fixed with 4% paraformaldehyde for 30 min at 4 °C. After washing with PBS, the slides were mounted with Dako cytomation fluorescent mounting medium before images were taken with a Zeiss LSM880 AiryScan confocal microscope. Images were acquired with an AiryScan detector in confocal mode with pinhole size 1 Airy unit (1AU). For image acquisition, Zen 2–3 (blue edition) software was used. Finally, images were possessed by Image J software (National Institute of Mental Health, Bethesda, MD, USA).

### 2.9. Statistical Analysis

Data are expressed as mean ± SD based on a minimum of three experiments unless otherwise indicated. Statistical analyzes were carried out with GraphPad Prism version 4 (GraphPad Software, Inc., La Jolla, CA, USA). Statistical significance was evaluated with the Student’s *t*-test or non-parametric ANOVA test.

## 3. Results

### 3.1. Characterization of the MS5-IR700 Conjugate

**Figure 1:**
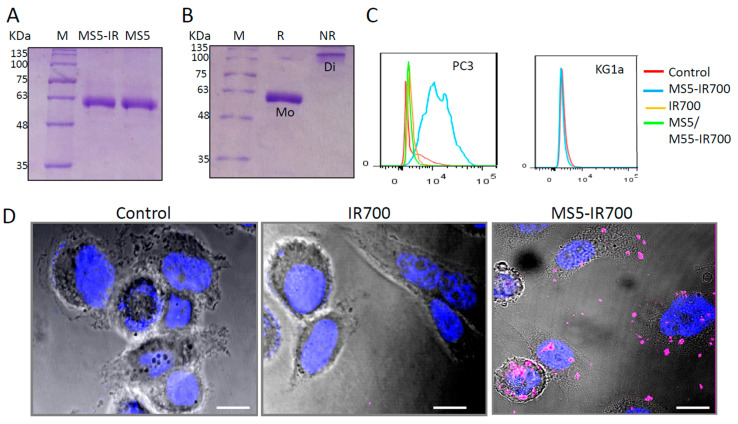
Characterization of the MS5-IR700 conjugate. (**A**) SDS-PAGE analysis of the MS5 minibody and MS5-IR700 conjugate. (**B**) SDS-PAGE analysis of the MS5-IR700 conjugate under reducing (R) and non-reducing (NR) conditions. Mo = monomers, Di = dimers, M = Markers. (**C**) Binding of the MS5-IR700 conjugate to PC3 prostate and KG1a cancer cell lines. The cells were stained with the conjugate (5 μg/mL) and then analyzed by flow cytometry. (**D**) Representative confocal images presenting PC3 cells stained with IR700 or MS5-IR700 conjugate (pink) following one-hour incubation at 37 °C. Nuclei were visualized with Hoechst 33342 staining (blue). Phase-contrast and fluorescence images were taken using a Zeiss LSM880 AiryScan confocal microscope. Scale bars present 20 μm.

The MS5 minibody recognizes a receptor overexpressed on the surface of cancer cells including prostate cancer, breast cancer, lung cancer, head and neck cancer, and various lymphomas derived from different stages of B cell differentiation [20]. Considering its potential universal use in targeted therapies, we evaluated its ability to deliver IR700 PS, a highly hydrophilic agent, to cancer cells. The MS5 minibody was conjugated to IR700 dye, purified, and then analyzed by SDS-PAGE. Under reducing conditions, the MS5-IR700 conjugate showed comparable molecular weight (55 KDa) as its unconjugated counterpart, suggesting that few IR700 molecules (1 to 2) were conjugated to each minibody molecule (Figure 1A). Under non-reducing conditions (NR), the MS5-IR700 conjugate was visualized as a major single band of 110 kDa, thus it would form disulfide bond-containing dimers under physiological conditions (Figure 1B). To confirm the binding to the PC3 prostate cancer cell line, flow cytometry was performed (Figure 1C). The MS5-IR700 showed enhanced binding to PC3 cells (blue histogram) relative to the control and cells stained with IR700 (red and orange histogram, respectively). Moreover, the binding to the cells was totally blocked with an excess of non-conjugated MS5 minibody (green histogram), suggesting specific binding. The KG1a cells do not express the MS5 receptor and therefore did not bind to the conjugate [20]. To probe the binding and internalization of the MS5-IR700 conjugate by PC3 cells, confocal microscopy images were generated (Figure 1D, as a representative example). The data confirm the flow cytometry data and demonstrate the internalization of the minibody-receptor complexes by PC3 cells as documented previously for the MS5 minibody [20]. In agreement with previous studies, the IR700 PS requires the antibody to bind target cells [9,15]. To date, most first and second-generation PSs studied for PDT enter both malignant and healthy cells leading to significant off-target effects [2]. In contrast, the IR700 offers the added advantage of being highly hydrophilic and therefore not taken up the cells as a free dye.

### 3.2. In Vitro Phototoxicity of the MS5-IR700 Conjugate

**Figure 2:**
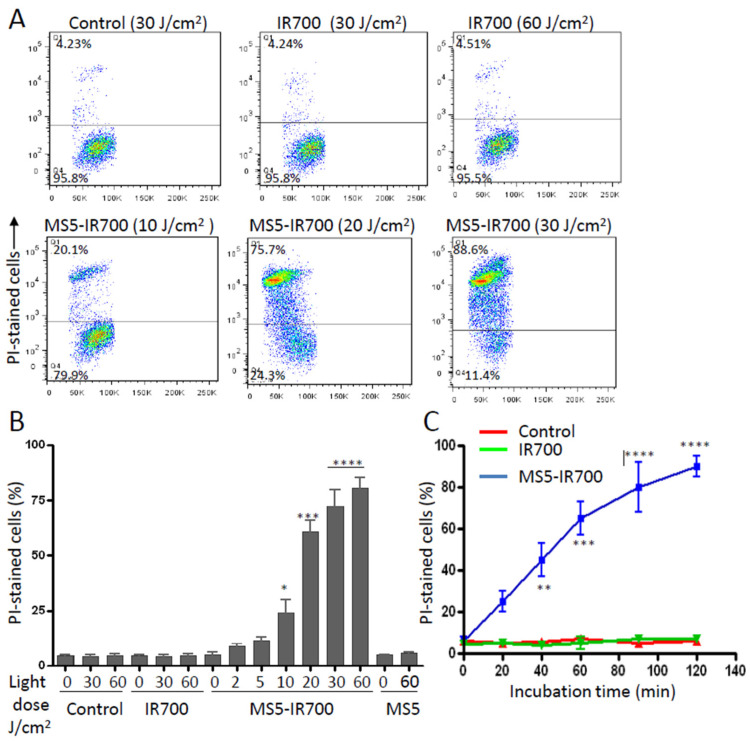
Quantitative cytotoxicity assay using flow cytometry. (**A**) Representative dot plots showing the effect of the MS5-IR700 conjugate on PC3 cell viability. The cells were incubated or not with the MS5-IR700 conjugate (5 μg/mL) for 1 h at RT, washed, and then exposed to various light doses at a power density of 17 mV/cm^2^. After 1 h incubation at 37 °C, they were collected with trypsin, stained with 1 μg/mL PI, and then analyzed by flow cytometry. As a control, the cells were also incubated with only IR700 (50 nM). The percentages of PI-stained cells are indicated. (**B**) The graph shows the percentage of live cells for each light dose. (**C**) Time course cell viability. Untreated and PC3 treated cells with IR700 (50 nM) or MS5-IR700 conjugate (5 μg/mL) were irradiated (35 J/cm^2^ at a power density of 17 mV/cm^2^) and then cell viability was quantitated at various time points using PI staining. The data represent the mean ± SD of three independent experiments. * *p* < 0.05, ** *p* < 0.01, *** *p* < 0.001, **** *p* < 0.0001.

**Figure 3:**
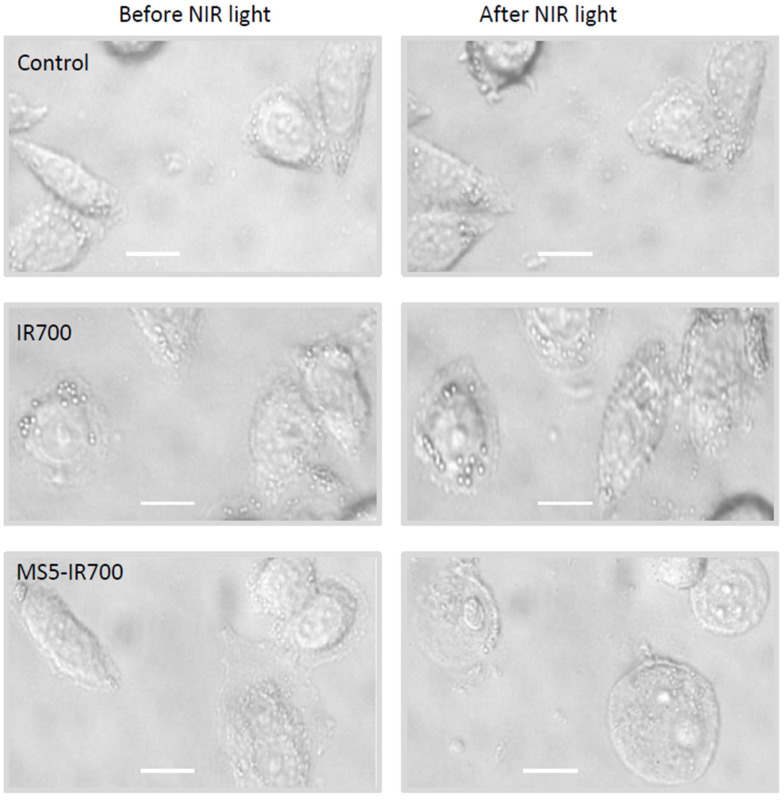
Cell morphology before and post-NIR light irradiation. Images presenting the morphology of PC3 cells incubated with IR700 (50 nM) or MS5-IR700 conjugate (5 μg/mL) before and after exposure to NIR light 35 J/cm^2^ at a power density of 17 mW/cm^2^. After irradiation, the cells were placed at 37 °C for 30 min and then the images were recorded. Of note, the cells have moved slightly from their initial locations after light irradiation. The images present morphologies that are representative of 5 independent wells. Magnification 40×. Scale bars present 20 μm.

First, we quantitatively evaluated the cell viability with flow cytometry and propidium iodide (PI) staining. PC3 cells were incubated with the MS5-IR700 (5 μg/mL) for 60 min at room temperature, washed, and then irradiated with increasing amounts of NIR light. Our preliminary experiments indicated that a concentration of 5 μg/mL of MS5-IR700 conjugate, which contains around 50 nM of IR700 dye associated with the minibody, is effective in killing prostate PC3 cells. As shown in Figure 2A,B, within one hour post-NIR light irradiation, the percentage of dead cells induced by the conjugate increased in a light-dose dependent manner, whereas no significant killing effect was observed with IR700 dye. Maximum cell death (more than 80%) was obtained at 30–60 J/cm^2^. Treatment of the cells with the MS5 minibody did not induce cell killing, thus the MS5 minibody alone is not harmless to the cells. Time-course analysis of cell viability using PI staining revealed a high percentage of cell death during the first hour in PC3 cells exposed to the conjugate with NIR irradiation (Figure 2C). Maximum membrane damage was peaked at 1.5 h after light irradiation. There was no significant difference in cell viability between the untreated and cells treated with the IR700 dye (50 nM). Additionally, we examined cell morphology 30 min post-NIR light irradiation (Figure 3). Untreated and cells treated with IR700 dye showed no obvious changes after light irradiation, while those treated with the MS5-IR700 conjugate displayed cellular swelling, round shape, and rupture of the cell and nuclear membranes after NIR light irradiation. Most of these changes were also observed within minutes of light exposure and they are often seen in necrotic cell death [22].

**Figure 4:**
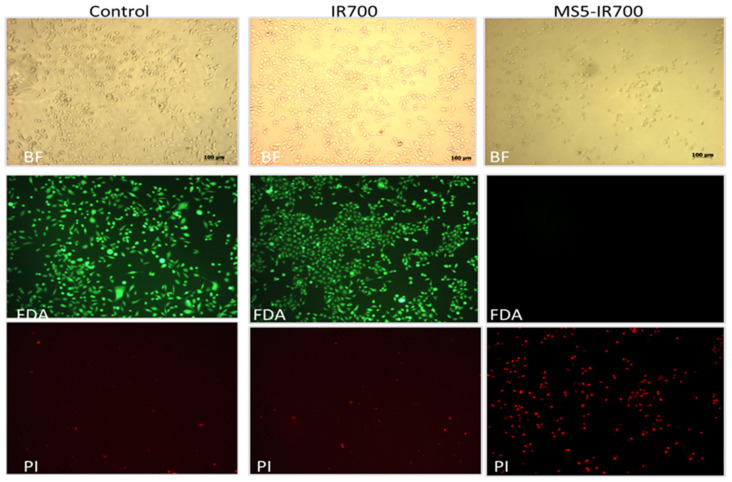
Cytotoxicity of the MS5-IR700 on PC3 cells. Experimental conditions are as in Figure 3. Light fluorescence microscopy images of cells counter-stained with FDA and PI were taken 6 h post-NIR irradiation. Scale bars present 100 μm. BF, bright field.

Next, the viability of PC3 cells was investigated using fluorescein diacetate (FDA) in combination with PI staining. PC3 cells were treated with the MS5-IR700 conjugate for one hour at RT, washed, and then exposed to NIR light (35 J/cm^2^). After 6 h incubation at 37 °C, the cells were stained with PI and FDA. Live cells convert the non-fluorescent FDA into the green fluorescent compound fluorescein, while compromised cells will emit red light, a sign of cell death. As shown in Figure 4 (last panel, PI staining), all cells treated with the MS5-IR700 conjugate were killed in comparison to those untreated or treated with IR700. Again, light microscopy images illustrate the severe alterations in the cell morphology post-NIR irradiation (upper panels).

### 3.3. Antibody Internalization Is Not Required for Cell Killing after NIR Light Irradiation

**Figure 5:**
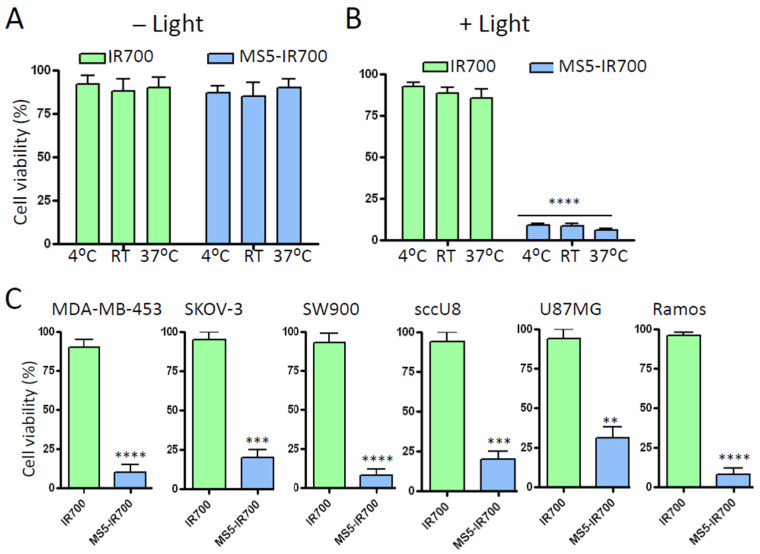
Cell killing does not require antibody internalization. (**A**) PC3 cells were incubated with IR700 (50 nM) or MS5-IR700 conjugate (5 μg/mL) at either 4 °C, RT or 37 °C for one hour. After washing, the cells were not (**A**) or exposed (**B**) to light (35 J/cm^2^ at a power density of 17 mW/cm^2^) and then incubated at 37 °C. After 16 h incubation, cell viability was measured by MTS assay. (**C**) Effects of the conjugate on other malignant cells. Conditions are as in A, except that the incubation with the test molecules was done at RT. Data are expressed as a percentage of the control cells (untreated) and are from triplicate determinations. They are representative of 3 independent experiments. ** *p* < 0.01, *** *p* < 0.001, **** *p* < 0.0001.

Next, we investigated whether the observed cytotoxicity requires the internalization of the MS5 minibody. The cells were incubated with the MS5-IR700 conjugate at 4 °C, RT, or 37 °C, washed to remove unbound molecules, and then irradiated (35 J/cm^2^). Cell viability was assessed 16 h post-NIR irradiation using MTS assay. As shown in Figure 5A, cells incubated with the MS5-IR700 conjugate did not show significant cell death (4% ± 2%) without light irradiation; however, NIR-irradiation killed 90% ± 5% of the living cells (Figure 5B). Cells incubated with the same concentration of free IR700 showed minimal cell death under light irradiation. Therefore, both MS5-IR700 and its excitation by NIR light are essential for cell killing. This observation provides additional proof that the ability of MS5-IR700 conjugate to kill cancer cells requires receptor binding. Of note, we observed no significant difference in cell killing efficacy whether the binding was performed at 4 °C, RT, or 37 °C prior to NIR light exposure, supporting that antibody internalization is not essential for the MS5-IR700 phototoxicity. In addition to PC3 cells, the conjugate also killed other malignant cells including breast MDA-MB-453, ovarian SKOV-3, lung SW900, head and neck scc-U8, Ramos lymphoma, and glioma U87MG (Figure 5C).

### 3.4. The Effect of the MS5-IR700 Conjugate Is Antigen-Specific

**Figure 6:**
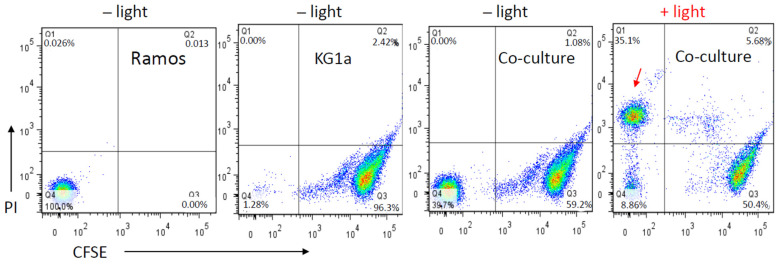
Cytotoxicity of the MS5-IR700 is receptor -dependent. KG1a cells were labeled with CFSE and then mixed with unlabeled Ramos cells (2 × 10^5^ each). After, the cells were incubated with the MS5-IR-700 conjugate (5 μg/mL) for one hour, washed to remove unbound molecules, and then exposed or not to NIR light (35 J/cm^2^ at a power density of 17 mW/cm^2^). After 6 h incubation at 37 °C, the cells were incubated with PI and analyzed with flow cytometry to check for dead cells. The arrow indicates PI-stained Ramos cells.

To confirm that the phototoxicity of the MS5-IR700 conjugate is antigen-specific, we co-cultured Ramos lymphoma cells, which bind to the MS5 minibody, with KG1a cells that do not bind [20]. KG1a cells were pre-labeled with carboxyfluorescein succinimidyl ester (green fluorescence), mixed with Ramos cells, and then incubated with the MS5-IR700 conjugate. After washing, the co-culture was exposed to NIR light (35 J/cm^2^) and then incubated at 37 °C for 6 h. As shown in Figure 6, the treatment only induced cell death in Ramos expressing the receptor for the MS5 minibody (last panel). Overall, the current NIR-PIT approach can combine the MS5 receptor binding and light activation of IR700 to enhance cancer specificity for the treatment of solid tumors or lymphomas.

### 3.5. Phototoxicity in Tumor Spheroids

**Figure 7:**
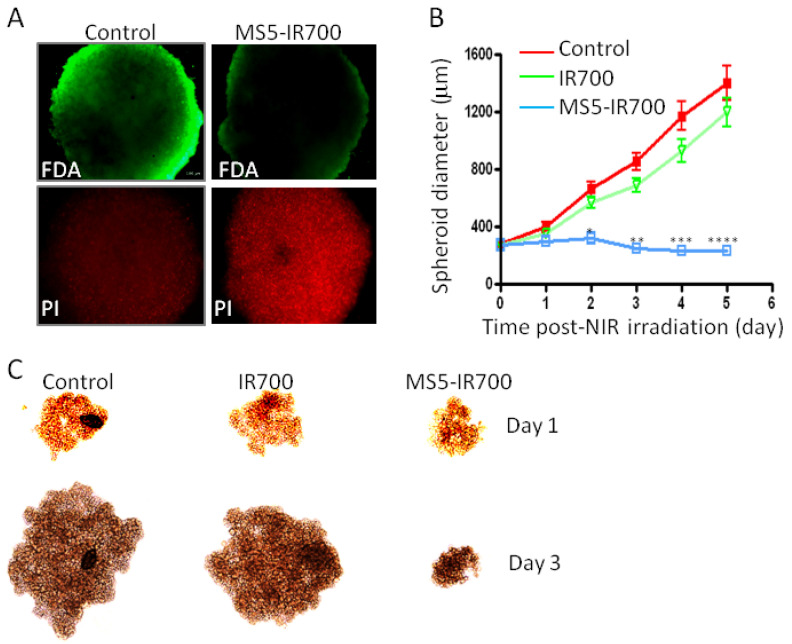
MS5-IR700 conjugate killed PC3 spheroids. (**A**) Spheroid around 200 μm diameters were incubated with the conjugate 10 μg/mL for 6 h at 37 °C and then irradiated at the light dose of 60 J/cm^2^ at a power density of 17 mW/cm^2^. After overnight incubation at 37 °C, the live/dead cells were visualized by incubation with FDA and PI. (**B**) Spheroid growth after NIR irradiation. Spheroids (around 350 μm in diameter) were incubated or not with the MS5-IR700 conjugate as in (**A**). A control group received IR700 (100 nM). After NIR exposure, spheroid diameters (3–4 spheroids/group) were measured every day up to 5 days. For illustration, light images of the spheroids on days 1 and 3 post-NIR irradiation are shown (**C**). The data are representative of 4 independent experiments. * *p* < 0.05, ** *p* < 0.01, *** *p* < 0.001, **** *p* < 0.0001.

After establishing that the MS5-IR700 conjugate could kill monolayer cells, its capacity to kill tumor spheroids, a model that mimics solid tumors [23], was evaluated. PC3 spheroids were treated or not with the MS5-IR700 conjugate for 6 h at 37 °C, as determined in early pilot experiments, and then they were irradiated with the 690 nm LED light at the light dose of 60 J/cm^2^. Twenty-four hours post-NIR light exposure, the cells were incubated with FDA and PI solutions and then imaged using an inverted Zeiss microscope. In agreement with the data from the monolayer cultures, the MS5-IR700-treated spheroids showed a pronounced cell death as compared to untreated spheroids (Figure 7A as a representative example, PI staining). We also investigated the effect of the MS5-IR700 conjugate on spheroid growth (Figure 7B,C). The treatment group receiving the MS5-IR700 showed a dramatic inhibition compared to the other two groups (*p* < 0.0001, day 5). As can be seen, when comparing the images in Figure 7C, the MS5-IR700 conjugate apparently inhibited spheroid growth even after one-day post-light irradiation.

### 3.6. Induction of Reactive Oxygen Species after PIT Treatment

**Figure 8:**
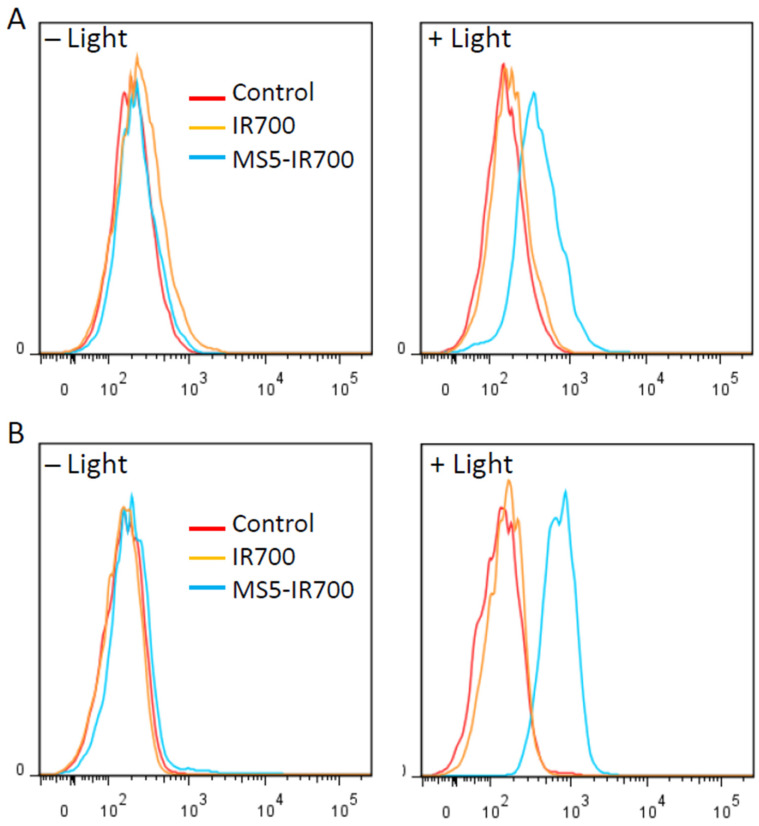
ROS induction. Cellular ROS levels were measured using the redox-sensitive indicator H2DCFDA. PC3 cells were labeled with H2DCFDA, incubated with the test molecules, washed, exposed to NIR light (35 J/cm^2^ at a power density of 35 mW/cm^2^), and then placed at 37 °C. After 6 (**A**) or 20 (**B**) h, the cells were analyzed by flow cytometry to detect fluorescent cells. Data are representative of 3 independent experiments.

The generation of reactive oxygen species (ROS) after irradiation usually contributes to the phototoxicity of PSs [3,9]. Therefore, we have investigated whether ROS production occurred during NIR-PIT using a cell-permeable fluorescent probe dichlorodihydrofluorescein diacetate (H_2_DCFDA). In the presence of ROS, H_2_DCFDA is oxidized and converted into a green fluorescent product that can be detected by flow cytometry. PC3 cells labeled with H_2_DCFDA (5 μM) and treated with the MS5-IR700 conjugate showed an increase in fluorescence at 6 and 20 h post-NIR irradiation, whereas those treated with IR700 displayed baseline levels of ROS production similar to untreated or MS5-IR700- treated cells without NIR irradiation (Figure 8A,B).

### 3.7. Surface Display of HSP70 and CRT after MS5-IR700 Treatment

**Figure 9:**
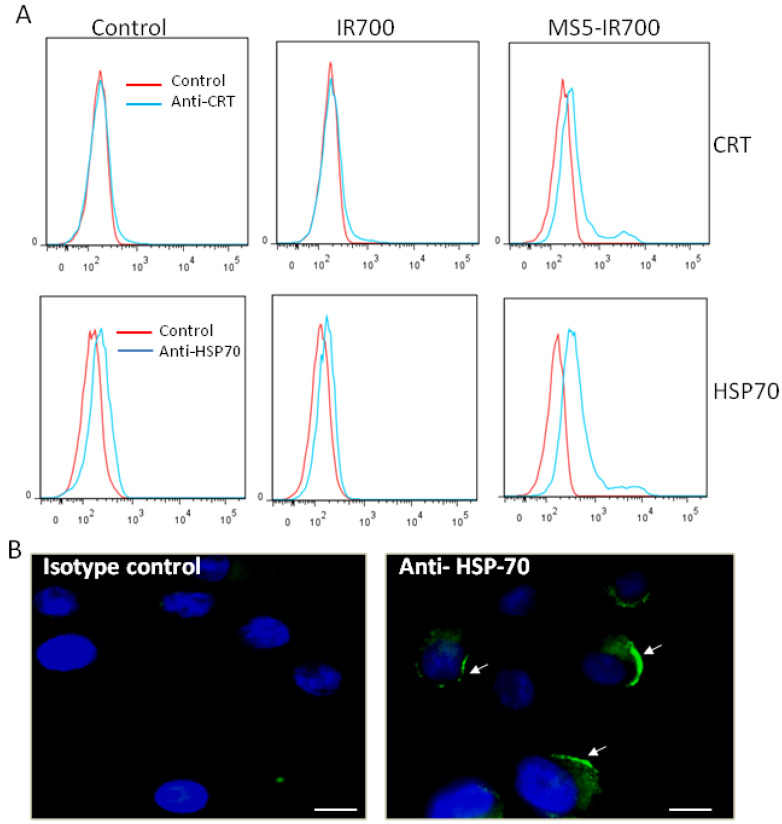
Surface display of heat shock protein 70 (HSP70) and calreticulin (CRT) after NIR light exposure. Experimental conditions are as in Figure 2. (**A**) Seven hours post-NIR irradiation, the expression of HSP70 and CRT on PC3 cells was investigated by flow cytometry using specific antibodies. The data are representative of 3 independent experiments. (**B**) Eight hours post-NIR light irradiation, the MS5-IR700-treated cells were stained with an anti-HSP70 antibody (green), fixed, and then fluorescence images were taken. The membrane location of HSP70 is indicated by arrows. Nuclei were visualized with Hoechst 33342 staining (blue). Scale bars present 20 μm.

Photodynamic therapy (PDT) often induces acute inflammation and recruitment of immune cells [24,25,26]. Such responses are triggered by the exposure or release of danger signals from the dying and damaged cells, so-called damage-associated molecular patterns (DAMPs) [25,27]. Among the expressed DAMPs, heat-shock protein 70 (HSP70) and Calreticulin (CRT) have been shown to be upregulated on the surface of target cells after PDT treatment [28,29,30,31]. Using flow cytometry, we investigated the surface localization of these two proteins 7 h post-NIR irradiation (Figure 9A). Compared to untreated and cells treated with IR700 dye, a significant increase in surface HSP70 and CRT was detected in cells treated with the MS5-IR700 conjugate. A small fraction of untreated cells displayed HSP70 on the surface although significantly lower than that seen in cells treated with the MS5-IR700 conjugate. In agreement with the flow data, fluorescence images acquired 8 h post-NIR light irradiation showed membrane display of HSP70 protein in MS5-IR700-treated cells (Figure 9B). The fluorescence signals for calreticulin were too weak to be imaged. Notably, flow cytometry is more sensitive than fluorescence microscopy.

## 4. Discussion

The use of antibody-photosensitizer conjugates or conceptually related innovative delivery strategies, which navigate the photosensitizing molecules to desired diseased cells and tissues, would enhance the efficacy and specificity of PDT. Here, we have evaluated the in vitro targeting potential of a novel minibody that recognizes a cell surface receptor expressed by a number of cancer cell lines. By covalently coupling the IR700 to the MS5 minibody, we have engineered a conjugate that efficiently killed PC3 prostate cancer cells in both cell monolayer and tumor spheroid cultures. Multicellular tumor spheroids are growing aggregates of cancer cells showing a transitional complexity between monolayer cells in vitro and solid tumors in vivo. Moreover, the treatment-induced cell surface display of HSP70 and CRT, suggesting the induction of immunogenic cell death, which is important for the induction of antitumor immunity that may lead to the destruction of distant cancer lesions. Under co-culture conditions, we also showed that cell death only occurs in cells that express the MS5 receptor and only in the presence of NIR and MS5-IR700 conjugate. The presence of the conjugate in the culture medium does not affect cell viability, supporting previous studies [9,13,14,15].

Although PDT is favorable in many ways, two key limiting factors prevent its implementation as a universal cancer treatment. First, most of the currently used PDT photosensitizers do not target tumors efficiently or avoid normal cells [3]. Second, typical PSs are most effectively excited by light with an approximate wavelength of 400 nm and, less efficiently by light in the 600 to 800 nm range [3]. To overcome the first challenge, PSs were conjugated to antibodies or antibody fragments against receptors expressed on target cells or tumor vasculatures [9,11]. The conjugates not only benefit from the high specificity of monoclonal antibodies but also the cytotoxicity initiated through ROS induction upon PS activation. Such a strategy known as PIT has been developed and tested successfully in several in vitro and in vivo cancer models [8,9,10,11,12,13,14,15,16]. Cetuximab, a humanized monoclonal antibody against epidermal growth factor receptor (EGFR) [32], has been linked to IR700 resulting in the generation of a conjugate that showed high selectivity and efficacy against various cancer types both in vitro and in vivo [9]. However, since EGFR is also expressed in normal epithelial tissue (e.g., skin and mucosa), targeting this receptor can lead to significant dermatologic and gastrointestinal toxicities [31]. With respect to other targets, Jing et al. showed that anti-CD133 antibody-conjugated to IR700 can target and kill malignant gliomas in vivo [33]. Among the cell surface markers, CD133 antigen is a highly promising target for cancer stem cells [34]. Similarly, the IR700-conjugated anti-cKIT antibody showed a significant anti-tumor effect against mouse gastrointestinal stromal tumors [35]. The conjugation of IR700 to rovalpituzuman, an anti- delta-like protein overexpressed by small cell lung cancer (SCLC) cells, led to the generation of a conjugate that effectively inhibited the growth of SCLC in mouse xenograft models after NIR irradiation [36]. Other PIT strategies targeting additional tumor markers also showed promising preclinical results in ovarian or pancreatic cancer [37,38,39,40].

Although the receptor for the MS5 minibody is not yet known, it should be considered an attractive target for PIT because it is overexpressed in many types of cancer that are located at a surface (e.g., skin cancer) or can be reached by an endoscope (e.g., prostate cancer, breast cancer). Prostate PC3 and Ramos lymphoma cell lines were used as models to study the killing efficacy and specificity of the MS5-IR700 conjugate in vitro. Other malignant cells were also killed by the MS5-IR700 conjugate, opening the possibility for its universal use. Notably, if the target antigen is common for all patients with various cancer types, the same antibody can be used for these patient groups, making the treatment widely applicable and perhaps cheaper.

Regarding irradiation wavelength, we have chosen the porphyrin-type PS IR700, a highly hydrophilic dye with peak absorption at 689 nm [9]. As an emerging PS, IR700 has attracted increased interest for its hydrophilicity, high photostability, and strong NIR absorption, allowing the treatment of deep tumors [3,11]. Additionally, this new agent is only active when bound to the target cells, while unbound molecules did not induce phototoxicity [9,11]. Based on the published data, free IR700 dye (e.g., 200 nM) did not bind to the cells or induce phototoxicity [9,40]. Therefore, cell death is dependent on the specific membrane binding of the antibody-IR700 conjugates. In line with these observations, we did not see significant phototoxicity with free IR700 (50 nM) on the tested malignant cell lines in the absence or presence of NIR light irradiation. Of note, after incubation, the cells were washed to remove unbound molecules prior to light exposure. However, no difference was found whether the cells were washed or not, indicating that free IR700 dye does not interact with the cell membrane. The binding to the cell membrane, and not just the presence of the conjugate or free IR700, is important to the phototoxicity of the conjugate.

With respect to the mechanism of cell death, several studies showed that the ROS that is generated by PDT or PIT can kill tumor cells directly via apoptosis and/or necrosis [3]. In the case of IR700, most of the performed studies with IR700-antibody conjugates concluded that necrosis is the main mechanism of cell death [9,11]. Recently, Ogawa et al. showed that NIR-PIT treatment using an antibody-IR700 conjugate can induce rapid membrane rupture after the rapid expansion of the cell volume [31]. And the water entering the cells was the cause of the observed swelling and rapid cell death. The observed morphology of PC3 cells 30 min post-NIR irradiation supports these findings. Notably, cells incubated with the MS5-IR700 conjugate and exposed to NIR light were killed within 30 min post-exposure. It should be noted that apoptosis is an active process and requires at least 2–3 h to occur [41]. Short-term cell viability assays showed that the MS5-IR700 conjugate was able to kill PC3 cells within 20–60 min after irradiation, whereas neither IR700 nor NIR-light irradiation alone affected cell viability. Moreover, the conjugate killed PC3 cells irrespective of whether the cells were incubated at 4 °C, RT, or 37 °C, indicating that the antibody internalization is not required for cell death. In addition to ROS that are known to trigger cell death during PDT, the mechanisms of cell death induced by the MS5-IR700 conjugate may involve additional mechanisms because at 4 °C the conjugate is mainly localized in the cell surface. Further studies are needed to elucidate the mechanism of cell death of MS5-IR700 conjugate, which is beyond the scope of this study.

By displaying low immunogenicity and secreting immunosuppressive factors, most solid tumors including prostate, breast, gliomas, ovarian and pancreatic cancers, are resistant to current immunotherapies [18,42]. A range of therapeutic approaches such as radiotherapy and PDT have been applied to induce immunogenic cell death (ICD). In addition to the direct killing of tumor cells, often PDT stimulates an immune response that mainly contributes to the killing of cancer outside the field of irradiation [39]. Tumor ICD is often triggered by the release of intracellular content including tumor antigens and DAMPs, which altogether trigger anti-tumor immune responses. Among the induced DAMPs, the display of HSP70 and CRT on the surface of cancer cells plays an important role in the induction of ICD [43]. Under physiological conditions, CRT is located in the endoplasmic reticulum and when it externalizes to the cell surface it functions as an “eat me” signal for macrophages and dendritic cells [44]. Ultimately, this process leads to the generation of tumor-specific cytotoxic T cells. Under our experimental conditions, the MS5-IR700 induced HSP70 and CRT on the surface of PC3 cells. Although we did not investigate the activation of immune cells by the MS5-IR700 killed cancer cells, their importance in tumor control is obvious and we will further expand on this topic in future in vitro and in vivo studies.

Monoclonal antibodies have been used to treat many different types of cancer including lymphoma, colorectal, prostate, and breast cancer [18,45]. Unfortunately, relapsed or refractory disease occurs in the majority of the patients and one of the main causes of resistance appears to be due to antigen loss [46,47]. Hence, new platforms for target and antibody discovery will facilitate the extension of targeted therapies to a broader array of cancers and overcome therapy resistance. We used several strategies such as immunoprecipitation and covalent cross-linking on live cells to help identify the MS5 binding receptor(s). Among the captured protein candidates, GRP78, vimentin, annexin 6, and certain integrins were identified. Some of these proteins such as GRP78 are overexpressed on the cell surface of most cancer cell types as compared to their normal counterparts [48,49,50]. However, we were unable to confirm the binding of the MS5 minibody to recombinant proteins. The receptor could be formed by the interaction between two or more protein partners expressed on the cell surface of cancer cells, yet to be identified.

## 5. Conclusions

Here, we describe a proof-of-concept study of molecular targeted photoimmunotherapy in cancer cells using a new MS5-IR700 conjugate. The developed conjugate induced cell death only in cancer cells that express the minibody receptor. The binding to the cell membrane, and not the presence of the conjugate or IR700 dye, is indispensable to the phototoxicity of the conjugate after NIR light irradiation. Although further preclinical studies are needed, the finding that the MS5-IR700 conjugate can kill various cancer cell types and induce ICD may provide treatment options for some patients who may not otherwise be candidates for current targeted PDT.

## Data Availability

Public data sources are listed in methods.
