# Correction: Sioud et al. Evaluation of In Vitro Phototoxicity of a Minibody-IR700 Conjugate Using Monolayer and Multicellular Tumor Spheroid Models. *Cancers* 2021, *13*, 3356

**DOI:** 10.3390/cancers13215513

**Published:** 2021-11-03

**Authors:** Mouldy Sioud, Petras Juzenas, Qindong Zhang, Andrius Kleinauskas, Qian Peng

**Affiliations:** 1 Division of Cancer Medicine, Department of Cancer Immunology, Oslo University Hospital, University of Oslo, Ullernchausseen 70, 0379 Oslo, Norway; Qindong.Zhang@rr-research.no; 2 Division of Laboratory Medicine, Department of Pathology, Oslo University Hospital-Radiumhospitalet, Ullernchausseen 70, 0379 Oslo, Norway; Petras.Juzenas@rr-research.no (P.J.); Andrius.Kleinauskas@rr-research.no (A.K.); Qian.Peng@rr-research.no (Q.P.)

The authors wish to make the following corrections to this paper [1]: In the original article, there was an error in Figure 4. The control panel (BF, FDA, and PI) was erroneously duplicated during the final figure assembly, resulting in two identical panels (control and IR700). The corrected Figure 4 appears below.


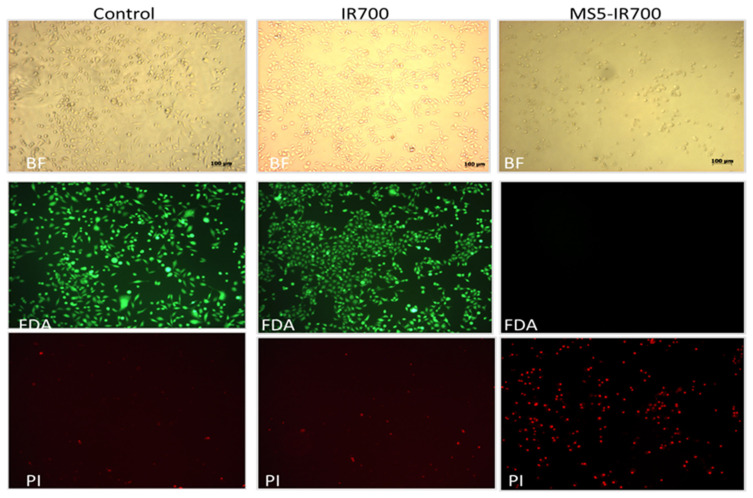


The authors apologize for any inconvenience caused and state that the scientific conclusions are unaffected. The original article has been updated.
